# Maternal Nutrition, Body Composition and Gestational Weight Gain on Low Birth Weight and Small for Gestational Age—A Cohort Study in an Indian Urban Slum

**DOI:** 10.3390/children9101460

**Published:** 2022-09-23

**Authors:** Raja Sriswan Mamidi, Santosh Kumar Banjara, Sridevi Manchala, Ch Khadar Babu, J. J. Babu Geddam, Naveen Kumar Boiroju, Bhaskar Varanasi, G. Neeraja, G. Venkat Raji Reddy, B. A. Ramalakshmi, R. Hemalatha, Gargi Meur

**Affiliations:** 1 Clinical Epidemiology Division, ICMR-National Institute of Nutrition, Hyderabad 500 007, India; 2 Clinical Division, ICMR-National Institute of Nutrition, Hyderabad 500 007, India; 3 Public Health Nutrition, ICMR-National Institute of Nutrition, Hyderabad 500 007, India

**Keywords:** birth weight, LBW, gestational age, SGA, maternal weight gain, body composition, dietary intake

## Abstract

Maternal nutritional status and care during pregnancy are essential for adequate birth weight. In this prospective cohort study (N = 1061) in an urban slum, we investigated the association of maternal anthropometry, body composition, gestational weight gain and dietary intakes with low birthweight (LBW, <2.5 kg). About one-third of the women were short (<150 cm), 35% were underweight (<45 kg), 23% suffered from chronic energy deficiency (CED, BMI < 18.5 kg/m^2^) and another 30% were overweight/obese. The mean age and BMI were 23 years and 21.7 kg/m^2^, respectively, and haemoglobin was 10.73 g/dL. The mean birthweight (N = 605) was 2.81 ± 0.5 kg, and the average gestational age was 38 ± 2 weeks. About 15% of infants had LBW, and 48% were small for gestational age (SGA). Maternal body composition was assessed by skinfold thickness (SFT) in all trimesters. In the first trimester (N = 762), we found that mean fat-free mass (FFM), fat mass (FM) and body fat percentage (% BF) were 38.86 kg, 11.43 kg and 21.55%, respectively. Low birthweight was significantly associated with preterm deliveries (*p* < 0.001) and less fat free mass (*p* = 0.02) in the third trimester. Among other factors were age (*p* = 0.017), maternal anthropometry (height: *p* = 0.031; weight: *p* = 0.059) and fewer antenatal check-ups (*p* = 0.037). Small size (SGA) was consistently associated with maternal bodyweight at all trimesters (term I, *p* = 0.013, term II, *p* = 0.003 and term III, *p* < 0.001), fat mass in the third trimester (*p* < 0.001) and maternal height (*p* = 0.003).

## 1. Introduction

Globally, an estimated 14.6% (20.5 million) children are born with low birthweight (LBW), and nearly half (47%) of those are in the south Asian region [1]. These children are at highest risk of infant mortality and are more likely to have poor childhood growth [2,3], lower analytical skills [4,5] and experience long term consequences of foetal reprogramming predisposing them to adulthood metabolic disorders like obesity, cardiovascular disease and diabetes [6,7]. Low birthweight increases the odds of wasting, stunting and being underweight in children up to 3.5-fold [8]. Birth weight is directly determined by two foetal factors: duration of gestation and rate of foetal growth [9]. Intrauterine growth restriction leads to small-size babies, found both in preterm and term births, and increases the risks of neonatal/infant mortality and poor growth [10,11,12]. Newborns weighing less than the 10th percentile of the usual weight for the sex and gestational age qualify as small for gestational age (SGA) [13,14]. Various factors like demographic, maternal, paternal anthropometrics, maternal metabolism and pre-conceptional nutritional status are correlated with foetal growth, but maternal variables like gestational weight gain are found to be the strongest predictors of birth weight [15,16].

Maternal nutritional status before and during pregnancy, and adequate pregnancy nutritional support are both critical for achieving optimal birthweight. The indicators of maternal nutrition pertaining to optimal foetal growth are pre-pregnancy body mass index (BMI), body composition and gestational weight gain (GWG) [17,18,19]. Gestational weight gain includes growth of fat mass (FM), fat-free mass (FFM), total body water (TBW), RBC mass, the foetus, the placenta, the amniotic fluid and all other products synthesized [18]. Early maternal weight gain reflects the adequacy of the nutrient supply to the placenta, which in turn ensures its adequate growth, development and function [20]. Likewise, excess GWG was found to increase the risk of large for gestational age babies [21]. Maternal BMI is a good predictor of birth weight, but it is only a surrogate indicator of nutritional status and not of maternal body composition. There are varied opinions on how maternal body composition rather than the total gestational weight gain correlated with birth weight. A substantial number of studies support that maternal fat-free mass, and not fat mass, has the strongest association with birth weight [22,23,24,25], but a few reports also found an association in total body fat (TBF) and TBW with neonatal birth weight [26,27]. The majority of the studies reflecting on maternal body composition as predictor of birth weight, however, were carried out with Caucasian women from Europe and North America; only a few were on East Asians [24]. These studies were conducted in the context of obesity, studied normal to obese subjects and focused on normal to excessive GWG [22,23,28]. However, total body fat and its distribution vary greatly among women of different ethnicity and, therefore, there is a serious lack of understanding on how maternal body composition and birth weight correlate in other ethnic populations. 

Measurement of body fat by segmental bioelectrical impedance analysis (BIA) was adopted by many hospital-based studies, but for field applications, anthropometrics and skinfold thickness are widely used to measure subcutaneous fat as a proxy for total body fat [29]. The methods for predicting adiposity from skinfolds by using Slaughter formulas were rigorously validated against dual energy X-ray absorptiometry (DEXA) and found to correlate well; they could predict cardiovascular risk factors in adolescents [30]. Although a single site may not reflect all fat stores, skinfold thicknesses are taken from multiple sites such as biceps, triceps, subscapular and suprailiac, indicating that subcutaneous fat on the limbs and body trunk are good predictors of FM. However, the skinfold thickness method lacks sensitivity in obese subjects, can vary with patterns of subcutaneous fat deposition in aging and differ between sexes [31]. Kulkarni et al. developed and validated many of the anthropometric prediction equations for the estimation of lean body mass and appendicular lean soft tissue in Indian men and women, using DEXA as a gold standard [32]. 

We conducted a longitudinal, prospective cohort study in a notified urban slum in India to understand the association of perinatal factors such as maternal anthropometry, body composition, GWG and dietary intakes with LBW and SGA. The slums are urban poverty pockets where the highest level of maternal and child undernutrition is recorded, especially among women and young children. Low birth weight and small size are the most prominent nutritional indicators of child undernutrition and are usually more prevalent in the slum population across the globe [33,34,35]. We have analysed associations between neonatal birth weight and some of the potential variables, focusing on LBW and SGA.

## 2. Materials and Methods

### 2.1. Study Design, Setting and Population

**Figure 1:**
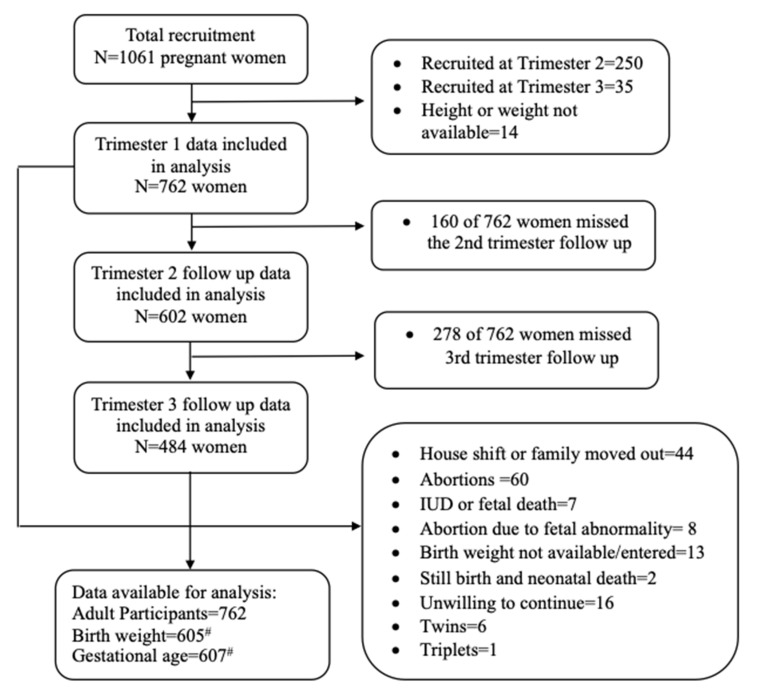
Flowchart of the participants in the longitudinal cohort study. For data analysis, only participants who were enrolled in Trimester 1 were considered (*n* = 776), of which 14 women had either height or weight missing and were excluded from final analysis as BMI could not be calculated. # Total number of children whose birth weight was available = 934. However, of the 762 women included in analysis, birth weight was available only for 605 infants, and gestational age was available for 607. Missing data accounted for subjects untraceable due to house relocation during delivery, intra-uterine death (IUD), neonatal death, termination of pregnancy, withdrawal from study, birth weight data not entered or not available as the participant went away to maternal home for delivery and multiple birth.

This longitudinal prospective cohort study was conducted in a notified slum in Hyderabad, India. The slum had about 5000 households and a population of about 25–30,000 as per census 2011, which has increased manyfold in the last decade. Pregnant women (*n* = 1061) were enrolled in the study by door-to-door survey of married women of child-bearing age in the slum, and women who were very early into pregnancy (~8–10 weeks) were enrolled. First date of last menstrual period (LMP) was recorded for calculation of gestational age. The survey was conducted by periodically visiting and recruiting subjects from at all four sections of the slum between March 2014 and July 2019. The details of the study participants and follow up are presented in Figure 1.

This study was conducted according to the guidelines laid down in the Declaration of Helsinki, and all procedures involving research study participants were approved by the Institutional Ethical Committee (Registration No. ECR/35/Inst/AP/2013) of Indian Council of Medical Research-National Institute of Nutrition (ICMR-NIN), Hyderabad. Written informed consent was obtained from all subjects.

### 2.2. Exposure Variables

#### 2.2.1. Socio-Demography

Data was collected using a pretested structured questionnaire. Socio-demographic and economic status, history of past pregnancy, knowledge attitude and practices of perinatal period were recorded. The family income was converted to USD based on average conversion rate in 2017–2018 (1 USD = INR 67). The subjects were enrolled at 8–10 weeks and followed up at 20–22 weeks, 28–30 weeks, 34–36 weeks and finally after delivery. Data on morbidity status, iron-folic acid supplementation and antenatal check-ups were recorded at every visit.

#### 2.2.2. Maternal Dietary Intake

Dietary intake of the participants was taken by 24 h dietary recall method on three different days at around 20 ± 2 weeks of gestation. Dietary macro and micro-nutrients were calculated using the reference values from Indian Food Composition Tables in IFCT 2017 [36]. Dietary adequacy was estimated by comparing consumption of food groups as well as nutrients intake with recommended amount for pregnant women [37].

#### 2.2.3. Maternal Anthropometry

Height and weight of all subjects were measured at first visit (~8 week of gestation) using a portable SECA scale (SECA robusta 813, Hamburg, Germany) to nearest 0.1 kg and a SECA height rod to nearest 0.1 cm (SECA 213 portable stadiometer). Gestational weight was monitored at every follow up, at 20–22, 28–30 and 34–36 weeks until delivery and soon after delivery. Mid-upper arm circumference (MUAC) and skin fold thickness at four sites—biceps, triceps, subscapular and suprailiac (BSF, TSF, SC, SI)—were measured at the first (8–10 weeks), second (20–22 weeks) and third (28–30 weeks) visit for body composition analysis. All measurements were performed in duplicate by two trained ANMs (auxiliary nurse midwife). MUAC was measured using a MUAC tape to nearest 1 mm and a Harpenden skinfold caliper (Baty International, Burgess Hill, West Sussex, UK) was used to measure skin-fold thickness to nearest 0.1 mm sensitivity. Maternal BMI (kg/m^2^) at 8–10 weeks gestation was considered baseline and graded according to the WHO’s Asia-Pacific standards [38]: Ranges used for BMI were <18.5, underweight; 18.5–22.9, normal weight; 23–27.5, overweight and ≥27.5, obese group. The ranges differed in WHO classification, where 18.5–24.9 is normal, 25–29.9 is overweight and ≥30 considered obese.

#### 2.2.4. Gestational Weight Gain and Body Composition Analysis

GWG was calculated by subtracting weight measured at first prenatal visit (8–10 weeks) from the last measured weight at 34–36 weeks. The lean mass or fat free mass (FFM) was calculated based on age, height, weight and skinfold thicknesses using the Equation (1) derived for female body composition by Kulkarni et al. [32].


Lean mass = 1.689 − (0.014 × age) + (0.120 × height) + (0.499 × weight) − (3.315 × logarithm of sum of 4 skinfolds)
(1)

Fat mass (FM) was calculated by subtracting lean mass from body weight and percent body fat was derived as percentage of weight. Gestational age for calculating the trimesters was as follows: Until 13 weeks 6/7 days (13 6/7) as first, 14 0/7 to 27 6/7 weeks as second, and 28 0/7 weeks and beyond as third trimester [39]. The FM (kg) was classified into three categories: low (<12.5), medium (12.6–16.5) and high (>16.5). FFM (kg) was also categorized into low (<47), medium (47–52.9) and high (>53) groups based on Aguirre et al. [28]. Likewise, % BF was categorized into three groups: low (<22.3 ± 5.1), medium (24.4–26.5 ± 5.0) and high (>26.6 ± 4.8) as per earlier studies [40].

#### 2.2.5. Maternal Anemia Status

A 5 mL venous blood sample was collected at the first visit to assess anaemia status. Haemoglobin was measured by cyanmethemoglobin method. In a sub-sample (*n* = 140), serum Ferritin was measured by ELISA (Calbiotech, El Cajon, CA, USA). Serum retinol was measured by HPLC (Dionex, Thermo Fisher, Waltham, MA, USA) to assess vitamin A status. Vitamin D status was assessed by measuring the serum concentration of 25-hydroxyvitamin D [25(OH)D] by HPLC. Serum zinc level was measured by atomic absorption spectrophotometry (AA 7000, Shimadzu, Kyoto, Japan). Sub-clinical level of inflammation was assessed by serum level of high-sensitivity C-reactive protein (HsCRP) using ELISA-based detection (ImmunoTag, St. Louis, MO, USA). Hepcidin is an iron-regulatory peptide hormone to indicate cellular iron stores. It is also a marker of recurrent infection and inflammation. Serum hepcidin level was measured by quantification of bioactive hepcidin 25 by ELISA (DRG Instruments GmbH, Marburg, Germany).

### 2.3. Birth Outcome

Delivery outcome and complications, date of delivery, neonatal birth weight and sex were obtained from hospital records upon visiting household soon or within 1 week after delivery. Two dependent variables analysed here were low birthweight and small size for gestational age. LBW was defined as newborns weighing less than 2.5 kg [41] and delivery before 37 weeks of gestation was preterm by standard nomenclature [42]. Gestational age of the newborn was calculated from LMP and date of delivery. Size of the newborn was estimated based on the older Canadian reference [13] as well as a recent international standard for newborn weight from INTERGROWTH-21st Project [14]. Based on these reference standards, newborns weighing less than 10th percentile for its gestational age were categorised as small for gestational age (SGA). The INTERGROWTH study, however, has the reference newborn weight available only from 33 weeks of gestation. Since our study had 12 infants born before 33 weeks of gestation, we opted for the Canadian standard for SGA analyses.

### 2.4. Statistical Analysis

All data were entered, and database was developed after duly performing range and consistency checks using Microsoft Excel. Descriptive statistics were carried out, and tables were generated for all the variables. Associations between potential categorical independent and dependent variables were tested using chi-square statistics. For quantitative variables, independent *t*-test and ANOVA were conducted. For non-parametric statistics, Mann–Whitney U-test was carried out. Logistic regression was used to assess relationships between perinatal factors (maternal age, BMI, education gestational age at delivery and neonatal sex) and birth outcomes (LBW and SGA). Unadjusted models and adjusted models were established, and odds ratio calculated. Outcomes were assessed using adjusted models based on prior epidemiological knowledge of their association or confounding effect from published literature (maternal age, education, BMI, neonatal sex, gestational age and family income). A *p* < 0.05 was considered statistically significant. All the statistical analyses were performed with SPSS 19.0 software (SPSS Inc., IBM, Chicago, IL, USA).

## 3. Results

### 3.1. Participants’ Characteristics

As shown in Table 1, the majority of the women were within 20–29 years (*n* = 645, 84.6%), and only 12% (*n* = 91) were pregnant at <20 years of age. About 40.6% women received over 12 years of education, and 96% (*n* = 706) of the women were homemakers at the time of recruitment. About 73% of the husbands received >10 years of education. Among the sampled families, 11% of the men were illiterate and 9% were casual laborers. Nearly half (48.2%) of the families had 4–7 members, and 60% (*n* = 455) had a monthly family income above ₹10,000, equivalent to $150. One-fifth (*n* = 150, 19.8%) of the families earned less than ₹7000 per month (about $105 pm). Anthropometric measurements of the subjects were recorded at first visit, i.e., 8–10 weeks into gestation, which showed that about one-third (32%) of the women were short statured (<150 cm) and about 35% were of low weight (<45 kg). About 23.2% were suffering from chronic energy deficiency (CED), while another 22.5% were overweight and 10% were obese as per Asian classification [38].

The mean age, height and weight of the subjects was 23 years, 152 cm and 50.3 kg, respectively (Table 2). The participants had a mean BMI of 21.7 kg/m^2^. Body composition analyses found mean lean mass of 38.86 kg, fat mass of 11.43 kg and 21.55% body fat. Haemoglobin (10.73 g/dL) and low serum ferritin (5.82 ± 1.57 ng/mL) levels indicated mild anaemia. The majority (81.1%) of the pregnant women had taken at least 100 tablets of the prophylactic IFA supplementation of 60 mg elemental iron and 500 g folic acid (IFA) during pregnancy. Among other biochemical parameters of maternal nutritional status which were measured, serum vitamin D level was 17.7(5.8) ng/mL, indicating deficiency, but serum zinc and retinol levels were adequate.

### 3.2. Neonatal Outcome

**Figure 2:**
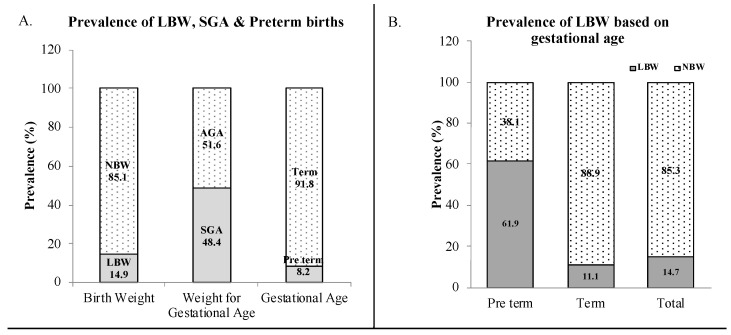
(**A**) Prevalence of low birth weight (LBW, <2.5 kg), small for gestational age (SGA, children in <10th percentile weight category for the gestational period) and preterm birth in the cohort population; (**B**) Prevalence of LBW among preterm (<37 weeks) and term newborn (n = 593).

Most women opted for hospital delivery, and nearly half of the newborn were delivered by C-section. Of the 762 women who were followed up from the first trimester, there were 7 cases of intra-uterine death (IUD), 8 cases of termination of pregnancy due to foetal abnormality or malformations and 1 case each of still birth and very premature birth, neonatal distress and death within 24 h. There were six cases of twin births and one triple birth, which were excluded from analyses. The mean (±SD) birth weight of the cohort’s children was 2.8(0.5) kg and gestational age was 38(2) weeks (Table 2). The overall prevalence of low birthweight was 14.9% (*n* = 90) and higher in females (18.1 vs. 12.3%). However, the size of newborns, compared to an old and well-accepted Canadian standard, found a 48.4% (*n* = 287) prevalence of SGA among all the cohort’s newborn infants, and SGA was higher among male children (50.3 vs. 46.1%). A very recent international standard for newborn weight derived from the INTERGROWTH-21st Project, which has representation of Indian reference as well, provided SGA prevalence of 32.4% (*n* = 188) among the cohort’s children. However, 12 infants born before 33 weeks could not be included in the calculation. The prevalence of LBW and SGA in full term babies was 11.1% and 49.5%, respectively. The overall prevalence of preterm birth (<37 weeks) was 8.2%, but about 60% of preterm infants had low birthweight (Figure 2).

### 3.3. Dietary Intake during Pregnancy and Birth Outcome

As shown in Table 3, the median intake of cereals and millets was much higher than the daily recommendation for Indians [37], while that for the other food groups, such as vegetables (~11%), roots and tubers (~30%), fruits (~50%) and pulses and legumes (66%) were considerably less. Consumption of flesh foods (26 g vs. 50 g per day), milk and milk products (230 vs. 400 mL per day) met only half the recommended requirement, while consumption of nuts, oils and seeds was less than 10%. All sources of protein combined contributed a little over two-thirds of the daily recommendation. In terms of nutrient intakes (Table 3), the median energy and protein intake seemed adequate as per the estimated average requirements (EAR) calculated for the Indian population, but because a major source of this protein was cereals, the quality of protein consumed may have been poor, and inadequate in lysine and other essential amino acids. The total fat intake was nearly double the recommended EAR. The median intake of micronutrients like thiamine, vitamin C and zinc was two-thirds or above, riboflavin was nearly one-third, niacin was three-quarters of the EAR. Dietary folate, vitamin A, iron and calcium were nearly half or less of the EAR. Dietary intake in terms of consumption of major food groups (Supplementary Materials Table S1) as well as energy and nutrients (Supplementary Materials Table S2) were similar among all women, irrespective of birth weight status of their infants. Only consumption of flesh foods was found to be negligible among mothers having LBW (0.24 g vs. 26.03 g) or SGA (11.2 g vs. 26.03 g) infants compared to their corresponding groups. However, these differences were not significant. Consumption of vegetables was comparatively higher in LBW (49.2 g vs. 31.2 g) and SGA (36.9 g vs. 30.4 g) groups, but even these differences were not significant between the corresponding groups. Categorization of the population into three tertiles based on high, medium and low intake of food groups (Supplementary Materials Table S3) failed to show any difference among the groups.

### 3.4. Infant Birth Weight Based on Socio-Demography, Maternal Anthropometry, Gestational Weight Gain and Body Composition

As shown in Table 4, LBW was associated with maternal age either less than 20 or more than (*p* < 0.05), preterm delivery (*p* < 0.001), fewer ANC visits (*p* < 0.05), shorter stature (*p* < 0.05) and a trend of maternal weight (*p* = 0.059), which was available only from early pregnancy (8–10 weeks). In the LBW category, 15.6% mothers were <20 years of age and 7.8% were above 30 years of age, compared to 11.7% and 2.5%, respectively, in the normal birthweight group. Among the LBW group, about 30% were born preterm compared to only 3.2% in NBW group. More women with short stature (40.9 vs. 29.4%) and low body weight (42.2 vs. 32%) were found in the LBW category. The mean maternal height was less by 1.23 cm (*p* = 0.057) in mothers of LBW children when compared to mothers of NBW children. The mean maternal weight was lower by 1.43 kg, 1.8 kg and 3.52 kg during the first, second and third trimester of pregnancy, respectively, in the LBW group (Table 5).

Tertiles of total gestational weight gain (GWG), gain in fat mass (FM), changes in fat free mass (FFM) or lean mass and increase in mid-upper arm circumference (MUAC) from Trimester 1 to 3 were compared between the women in the LBW and NBW groups, but the difference was not significant (Table 4). Between the first and third trimester, FFM decreased in all women and the loss was higher by 1.87 kg in the LBW group. In the same period between the first and third trimester, FM increased in all women, but it was lower by 0.67 kg in the LBW group. However, the percentage of body fat (PBF) in the LBW group was higher by 2.13% points compared to the NBW group. There was a marginal increase in weekly gain in FM and % BF in mothers with LBW children over NBW. None of these differences were significant. There were more women with lower GWG during the trimesters (1st tertile: 41.3 vs. 31.2%) among the LBW compared to AGA group, but this difference was not significant. Birth weight was strongly associated with total weight (*p* = 0.01) and FFM (*p* < 0.05) in the third trimester, but not with FM and weekly total GWG or gain of FM and FFM. MUAC was not a predictor of birth weight at any stage of pregnancy.

### 3.5. Small for Gestational Age Based on Socio-Demography, Maternal Anthropometry, Gestational Weight Gain and Body Composition

As shown in Table 4, SGA was significantly (*p* < 0.05) associated with maternal height and weekly weight gain in FFM during the trimesters. More women were shorter in the SGA group (37.3 vs. 25.8%, *p* = 0.003). The mean maternal height was 1.37 cm lower in the SGA category over the AGA category (Table 5). In addition, more women had low body weight (36.9 vs. 31%), although this difference was not significant. There were more women who had lowest GWG during the trimesters (1st tertile: 37.2 vs. 27.5%) among the SGA group compared to the AGA group.

It was observed that the mean maternal weight was 2.1 kg, 2.58 kg and 3.8 kg less during first, second and third trimesters, respectively, for the SGA group, and these differences were significant. All indicators of maternal body composition, FFM, FM and PBF were significantly lower in the SGA group by 950 g, 1.15 kg and 1.36% in the first trimester, compared to the AGA group. In the third trimester, FFM, FM (*p* = 0.002) and % BF were lower by 0.77 kg, 3.03 kg and 1.8% points. There were more women in the lowest tertile for weekly gain of FFM during the trimesters (1st tertile: 36.4 vs. 29.8%) among those in the SGA group compared to the AGA group. The weekly gain of FM, PBF or MUAC was not different in the SGA group during the first and third trimesters. The mean birth weight was less by 630 g in SGA infants. We did not find a difference among the groups (LBW vs. NBW and SGA vs. AGA) in terms of family income, maternal occupation, maternal anaemia status and iron deficiency or in adherence to 100 days of IFA supplementation.

### 3.6. Regression Analysis for Predictors of LBW and SGA

Logistic regression analysis (Table 6) found significant association of preterm delivery, female sex, less education, mother’s chronic energy deficiency as higher risk and age between 20–29 years as lower risk for low birth weight in the unadjusted model. However, after adjusting for mother’s age, family income, education and BMI, only neonatal sex and preterm births came out as significant predictors. For the SGA group, however, neither crude nor adjusted models showed any significant associations with the same predictors. Maternal dietary intake, IFA supplementation, hemoglobin and other nutritional markers such as vitamin A, D, serum ferritin, serum zinc and inflammatory markers (hepcidin and hsCRP) were not found to be significantly associated with low birth weight or small for gestational age in the bivariate analyses and, therefore, these parameters were not included in the final model.

## 4. Discussion

In our study population from an urban slum, 14.7% babies were born with low birthweight among all live births, which is very similar to the global average of 15%. A recent review identified a global trend of a ~1% yearly decline of LBW, based on data from country surveys in 2000 and later years [43]. However, 49.5% prevalence of SGA in full-term babies (overall 48%) in our population, as derived using the Canadian standard, was very high compared to the estimated SGA of 27% in all live births in low- and middle-income countries in 2010 [44]. While the Canadian reference standard for newborn weight was always used by us in our earlier studies in this population [45], if it was replaced with a more recent international standard for newborn weight, derived from the INTERGROWTH-21st Project, a lower SGA prevalence of 32.4% was found, but even this was considerably higher compared to the global SGA prevalence. Globally, South Asia and Africa carry the highest burden of SGA newborns. In 2010, the largest number of SGA births in the world was reported from India, accounting for 12.8 million (uncertainty range of 11.5–14.3 million), and accounting for nearly 46.9% of live births [46]. While SGA also contributes to the LBW pool of neonates, there are other concerns, such as intra-uterine growth retardation, that dominate a large number of pregnancies in the poor socio-economic stratum. In our study population, 23.2% of women were chronic energy deficient at the time of conception, although 22.5% were overweight and 10% were obese. According to the most recent National Family Health Survey 5 [47], prevalence of CED and overweight/obesity among pregnant women in Hyderabad’s urban population was reported to be 12.4% and 51%, respectively. It is clear that higher CED prevalence indicated the severity of undernutrition among women living in the slums.

It has been shown by many studies that neonatal birth weight is closely associated with maternal lean mass [19], increase in maternal body mass index (BMI) and GWG, all of which reflect the nutritional status of the mother throughout pregnancy. Our analyses of the maternal indicators identified early pregnancy weight, which is closest to preconception weight, and pregnancy weight gain as strong predictors of infant birthweight. The total average weight gain during the period of gestation was estimated to be around 7.24 kg for an average difference of 20 weeks (range: 15–33 week), while the mean weight gain during the trimesters among the LBW and NBW categories were 5.4 and 7.49 kg, respectively. The ideal pregnancy weight gain recommended for Indian women during first, second and third trimesters is 119 g/week, 420 g/week and 378 g/week, respectively, constituting a required fat mass gain of 36.4 g/week, 132.3 g/week and 118.3 g/week during the first, second and third trimesters, respectively, and a total fat deposition of 3.69 kg during the trimesters, which ideally should contribute to a total weight gain of 10–12 kg during the gestation period [37]. In the present study, the average weight gain during the first to third trimesters was 362 g/week with wide variability (range: 30–930 g/week). Our findings corroborated earlier reports on the most common associations of birth weight with maternal factors such as the mother’s height and weight, age, better antenatal care, and gestational age at delivery, as preterm births are often associated with LBW. Maternal body composition analyses found that both total body weight and FFM in Trimester 3 was significantly associated with birth weight, while GWG and FM did not come out as a strong predictor, as other studies also reported. On the contrary, SGA was not only associated with the mother’s height, but also with weight in every trimester and with maternal body composition, including FM, FFM and PBF during the first trimester, and FM in the third trimester. The pathophysiology of SGA lies in the uterine growth reduction and affects all, including term births. This is not same in LBW, as ~30% infants are preterm born. However, MUAC gain and weekly GWG did not come up as predictors of LBW and SGA. Our observation points at pre-conceptional nutritional status, perinatal nutritional support and pregnancy care as important to set the optimum rate of foetal growth. As our study indicated that maternal body composition was more significantly associated with size for gestational age than with birthweight, it implies that both lean mass and fat mass at early and late pregnancy are important for women to avoid small size babies, especially in populations where a higher proportion of women are undernourished.

Although the association between dietary intake and birth outcomes could not be established in the present study due to lack of dietary data from multiple time points, it is needless to emphasize the importance of maternal nutrition for adequate weight gain before and during pregnancy. Comparison of present findings with an earlier study conducted a decade ago in the same study area revealed a positive trend in some of the indicators [48]. The mean maternal age improved by one year and maternal weight improved by 3.8 kg, although gestational age lowered by 0.7 weeks. The number of ANC visits improved by 1.5, and the mean birth weight improved by 160 g. The LBW prevalence among full-term newborns declined by 6.4% between the two study periods in the same community, with the earlier prevalence measured at 17.5%. There was overall improvement in the indicators pertaining to nutritional status of women and infants, which could be attributed to multiple governmental interventions, including *Poshan Abhiyaan* by the central government.

The strength of the study is the availability of a large sample size, a longitudinal design instead of a cross-sectional study, follow up data on anthropometrics and skinfold thickness for body composition analyses as opposed to BMI, which is an indicator of obesity, but a poor indicator of body composition, i.e., FFM and FM. Another strength of our study is that we have new insight on the association of maternal body composition with suboptimal birth outcome (LBW and SGA) in a population in which undernutrition is high, as opposed to reports from obese mothers with large babies. In addition, more studies reported on the Caucasian population from Europe and North America, which predominates our current understanding of maternal body composition in relation to birth weight, and far less representations are from other ethnicities [15,22,23,27]. Because body composition varies closely with ethnicities [47], this study addresses the gap to fill our understanding on Indian women from underprivileged communities.

There are, however, a few limitations in this study, such as a lack of follow up data on dietary intake and anaemia status at all trimesters, as the women were taking IFA supplementation which had supposedly improved their anaemia status. We did not include parity in our analyses, which is known to affect maternal FM as residual from previous pregnancies. Another limitation of the study was that we used skin fold thickness for body composition instead of other precise methods such as BIA and DEXA, as these methods were not recommended for use in pregnant women. While skinfold thickness correlates well with subcutaneous fat distribution, a limitation of SFT is that it cannot predict fats deposited around internal organs and in visceral obesity, and therefore is not suitable for measuring obese subjects.

## 5. Conclusions

This study reveals the importance of better maternal anthropometrics and body composition before and during pregnancy for improved birth outcome. Among the modifiable variables to improve LBW are better pre-conceptional weight, higher fat free mass and a greater number of ANC visits. SGA is a consequence of intrauterine growth retardation due to many possible factors, but is influenced by maternal height, optimum weight at start and gain throughout pregnancy. Maternal FM at the first and third trimester turned out to be an important determinant for optimal neonatal size, which is applicable to women on the undernutrition side of the spectrum. Maternal factors are the key drivers for optimum birth outcome, underscoring the need for adequate maternal nutrition and care during pregnancy.

## Figures and Tables

**Table 1:** Socio-demographic and maternal characteristics.

Particulars	Total N	Categories	*n* (%)
Age at enrolment (year)	762	<20	91 (11.9)
		20–29	645 (84.6)
		≥30	26 (3.4)
Education	762	Illiterate	53 (7.0)
		Primary (7 year)	115 (15.1)
		Secondary (>10 year)	285 (37.4)
		Higher (>12 year)	309 (40.6)
Occupation of the pregnant women	731	Homemaker	706 (96.6)
		Others	25 (3.4)
Husband’s education	762	Illiterate	89 (11.7)
		Primary	110 (14.4)
		Secondary	306 (40.2)
		Higher	257 (33.7)
Husband’s occupation	762	Labour	66 (8.7)
		Business	60 (7.9)
		Others	636 (83.5)
Family size	761	≤3	323 (42.4)
		4–7	367 (48.2)
		≥8	71 (9.3)
Monthly family income (Rs)	756	<7000	150 (19.8)
		7000–10,000	151 (20.0)
		>10,000	455 (60.2)
Maternal Characteristics			
Short stature (<150 cm)	703	<150	240 (32)
		≥150	510 (68)
Lower bodyweight (<45 kg)	742	<45	265 (34.8)
		≥45	497 (65.2)
BMI Asian classification	750	CED (<18.5)	174 (23.2)
		Normal (18.5–22.99)	332 (44.3)
		Overweight (23–27.49)	169 (22.5)
		Obese (≥27.5)	75 (10)
IFA taken for 100 days	613	Yes	497 (81.1)
		No	116 (18.9)
Place of delivery	704	Home	5 (0.81)
		Hospital	609 (99.19)
Type of delivery	607	Caesarean section	289 (47.61)
		Normal	318 (52.39)

**Table 2:** Mean and median values of maternal anthropometry, nutritional status and newborn parameters.

Maternal Indicators	N	Mean	SD	Median	P25–P75
Age (year)	762	23	3.2	23	21–25
Mother’s height (cm)	750	152.16	5.76	152	148.4–156
Mother’s weight (kg)	762	50.29	10.49	48.8	42.7–55.9
Haemoglobin (g/dL)	742	10.73	1.52	10.9	9.8–11.79
Ferritin (ng/mL)	128	5.82	11.57	0.49	0.17–5.53
Zinc (µg/dL)	124	79.5	25.7	75.6	60.2–92.3
Vitamin D (ng/mL)	115	17.7	5.8	17.2	13.6–21.4
Vitamin A (µg/dL)	115	53.9	14.3	53.2	43.5–61.2
FFM (kg)	762	38.86	5.6	38.43	35.41–41.82
FM (kg)	762	11.43	5.98	10.41	6.96–14.47
BF%	762	21.55	7.81	21.31	16.43–25.84
BMI (kg/m^2^)	750	21.71	4.28	20.93	18.71–24.09
Newborn Parameters					
Neonatal birth weight (kg)	605	2.81	0.52	2.80	2.50–3.12
Gestational age at delivery (weeks)	607	38	2	38	38–40

FFM, fat free mass; FM, fat mass; BF, body fat; BMI, body mass index; SD, standard deviation.

**Table 3:** Median intake of food groups and nutrients among pregnant women based on % of balanced diet requirement for the Indian population.

Parameters	Median	IQR	* Recommendation	% of Recommended Amount
Food groups				
Cereals and millets (g)	374.53	232.70	325	115.23
^#^ Pulses and legumes (g)/ Meat, poultry, fish and sea foods (g)	59.67	94.56	90	66.3
Fats and edible oils (g)	21.35	19.52	25	85.4
Milk and milk products (mL)	230.00	252.28	400	57.5
Nuts and oil seeds (g)	2.96	16.62	40	7.4
Vegetables (g)	32.77	86.33	300	10.9
Roots and tubers (g)	30.00	48.14	100	30
Fruits (g)	75.74	116.08	150	50.46
Dietary nutrients				
Energy (Kcal)	2408.02	1219.72	2480	97.1
Protein (g)	64.61	37.59	54	119.6
Total fat (g)	55.77	38.64	30	185.6
Thiamine (mg)	1.06	0.65	1.6	66.25
Riboflavin (mg)	0.89	0.50	2.3	38.7
Niacin (mg)	10.56	6.51	14	75.42
Dietary folate	196.33	147.70	480	40.89
Vitamin C (mg)	45.3	45.71	65	69.7
Vitamin A (µg)	201.45	219.29	406	49.6
Iron (mg)	10.09	6.82	21	48.04
Zinc (mg)	8.03	4.81	12	66.9
Calcium (mg)	502.72	368.62	800	62.84

IQR, inter-quartile range. ^#^ About 30 g of pulses can be replaced with 50 g of flesh food. * Recommendations of food groups for meeting a balanced diet for Indians are according to 2020 recommendations, but recommendations for nutrients are based on estimated average requirement.

**Table 4:** Infant birthweight based on maternal anthropometry, gestational weight gain and body composition.

Maternal Indicators		Birthweight						*p*	Neonatal Size						*p*
		LBW (<2.50 kg)		NBW (≥2.50 kg)		Total			SGA		AGA		Total		
		*n*	%	*n*	%	*n*	%		*n*	%	*n*	%	*n*	%	
Age at enrolment (years)	<20	14	15.6	60	11.7	74	12.2	0.017	35	12.2	36	11.8	71	12	0.849
	20–29	69	76.7	442	85.8	511	84.5		244	85	259	84.6	503	84.8	
	30 & above	7	7.8	13	2.5	20	3.3		8	2.8	11	3.6	19	3.2	
	Total	90	100	515	100	605	100		287	100	306	100	593	100	
Gestational age (weeks)	<37	26	29.9	16	3.2	42	7.1	<0.001	15	5.2	28	9.2	43	7.3	0.066
	≥37	61	70.1	490	96.8	551	92.9		272	94.8	278	90.8	550	92.7	
Hb group (g/dL)	Anaemic (<11)	50	55.6	256	50.6	306	51.3	0.385	153	51.5	145	50.7	298	51.1	0.844
	Non-anaemic (≥11)	40	44.4	250	49.4	290	48.7		144	48.5	141	49.3	285	48.9	
MUAC_T1-T3 gain (cm)	1st tertile (≤−0.999)	10	18.5	62	19.5	72	19.4	0.641	34	19.1	34	18.1	68	18.6	0.854
	2nd tertile (0–1.19)	26	48.1	132	41.5	158	42.5		79	44.4	80	42.6	159	43.4	
	3rd tertile (≥1.2)	18	33.3	124	39	142	38.2		65	36.5	74	39.4	139	38	
FFM_T1-T3_weekly gain (kg)	1st tertile (≤−0.764)	24	37.5	129	33.2	153	33.8	0.778	80	36.4	67	29.8	147	33	0.05
	2nd tertile (−0.765–−0.516)	21	32.8	132	33.9	153	33.8		63	28.6	89	39.6	152	34.2	
	3rd tertile (≥−0.517)	19	29.7	128	32.9	147	32.5		77	35	69	30.7	146	32.8	
FM_T1-T3_weekly gain (kg)	1st tertile (≤0.812)	17	26.6	133	34.3	150	33.2	0.217	79	35.9	70	31.3	149	33.6	0.558
	2nd tertile (0.813–1.113)	27	42.2	122	31.4	149	33		69	31.4	78	34.8	147	33.1	
	3rd tertile (≥1.2)	20	31.3	133	34.3	153	33.8		72	32.7	76	33.9	148	33.3	
GWG_T1-T3 (kg)	1st tertile (≤0.287)	26	41.3	119	31.2	145	32.6	0.99	80	37.2	61	27.5	141	32.3	0.79
	2nd tertile (0.288–0.415)	23	36.5	128	33.5	151	33.9		71	33	79	35.6	150	34.3	
	3rd tertile (≥0.416)	14	22.2	135	35.3	149	33.5		64	29.8	82	36.9	146	33.4	
ANC visits	<4	3	3.4	4	0.8	7	1.2	0.037	4	1.4	3	1	7	1.2	0.649
	≥4	84	96.6	492	99.2	576	98.8		275	98.6	292	99	567	98.8	
Mother’s height (cm)	<150	36	40.9	149	29.4	185	45.1	0.031	106	37.3	77	25.8	183	45.8	0.003
	≥150	52	59.1	358	70.6	410	54.9		178	66.7	222	74.2	400	54.2	
Mother’s weight (kg)	<45	38	42.2	165	32.0	203	49.3	0.059	106	36.9	95	31.0	201	51.3	0.130
	≥45	52	57.8	350	68	412	50.7		181	63.1	211	69.0	392	48.7	

Groups were compared based on categories using chi-squared test. FFM, fat free mass; FM, fat mass; MUAC, mid-upper arm circumference; ANC, antenatal check-ups; first, second and third tertile represent low, medium and high categories; GWG, gestational weight gain.

**Table 5:** Birthweight based on mean maternal anthropometric indicators and body composition.

Maternal Indicators	Birth Weight (kg)						*p*	Neonatal Size						*p*
	LBW (<2.50)		NBW (≥2.50)		All			SGA		AGA		All		
	Mean	SD	Mean	SD	Mean	SD		Mean	SD	Mean	SD	Mean	SD	
Age (years)	23.4	3.9	23	2.9	23	3.1	0.267	23	3.1	23.1	3.1	23	3.1	0.935
Height (cm) (*n* = 595)	151.14	6.04	152.37	5.49	152.19	5.59	0.057	151.48	5.55	152.85	5.52	152.18	5.57	0.003
T1 weight (kg) (*n* = 605)	49.18	11.57	50.61	10.17	50.39	10.39	0.229	49.19	9.63	51.29	10.88	50.27	10.34	0.013
T2 weight (kg) (*n* = 525)	51.75	10.93	53.55	9.77	53.29	9.96	0.14	51.9	8.93	54.48	10.68	53.19	9.92	0.003
T3 weight (kg) (*n* = 453)	54.58	10.45	58.1	10.02	57.6	10.14	0.01	55.61	8.74	59.41	11.2	57.53	10.23	<0.001
T1 MUAC (cm) (*n* = 605)	24.56	3.96	24.73	3.46	24.7	3.54	0.676	24.45	3.52	24.86	3.57	24.66	3.54	0.16
T2 MUAC (cm) (*n* = 145)	24.47	2.33	24.51	2.86	24.51	2.79	0.955	24.51	2.65	24.45	2.95	24.48	2.8	0.902
T3 MUAC (cm) (*n* = 453)	25.11	3.33	25.45	3.29	25.4	3.3	0.44	25.09	3.11	25.67	3.5	25.38	3.32	0.067
HB (g/dL) (*n* = 596)	10.67	1.56	10.78	1.5	10.76	1.51	0.519	10.72	1.62	10.82	1.41	10.77	1.52	0.416
Serum ferritin (ng/mL) (*n* = 115)	8.33	14.88	5.17	9.50	5.81	10.79	0.210	5.94	10.28	5.35	10.93	5.60	10.62	0.773
Serum zinc (µg/dL) (*n* = 112)	79.3	21.1	80.5	27.1	80.3	25.9	0.841	79.8	27.1	80.4	24.9	80.2	25.7	0.904
Serum vitamin D (ng/mL) (*n* = 102)	17.0	6.1	17.6	5.8	17.5	5.9	0.694	17.8	6.2	17.2	5.7	17.4	5.9	0.605
Serum hsCRP (mg/L) (*n* = 115)	1.69	1.77	1.78	2.06	1.76	2.00	0.844	2.07	2.44	1.57	1.62	1.78	2.01	0.198
Serum hepcidin (ng/mL) (*n* = 110)	55.76	51.23	74.77	72.03	70.97	68.59	0.247	79.78	79.87	65.38	59.86	71.38	68.92	0.286
Newborn parameters														
Gestational age (weeks) (*n* = 594)	37	2	39	1	38	2	<0.001	2.49	0.32	3.12	0.45	2.82	0.5	<0.001
Maternal Body Composition														
FFM_T1 (kg)	38.02	6.56	39.04	5.43	38.89	5.61	0.11	38.34	5.01	39.29	6.06	38.83	5.59	0.039
FM_T1 (kg)	11.16	6.28	11.56	5.84	11.5	5.9	0.55	10.85	5.48	12	6.22	11.44	5.9	0.017
BF_T1 (%)	21.49	8.55	21.76	7.61	21.72	7.75	0.761	20.95	7.29	22.31	8.19	21.65	7.79	0.034
FFM_T2 (kg)	35.57	9.11	33.54	9.87	33.77	9.75	0.561	34.03	9.88	33.51	9.75	33.77	9.75	0.81
FM_T2 (kg)	15.82	8.11	18.97	9.53	18.62	9.39	0.347	18.16	8.92	19.06	9.9	18.62	9.39	0.668
BF_T2 (%)	30.67	16.18	35.98	17.35	35.4	17.21	0.385	34.73	17.23	36.03	17.37	35.4	17.21	0.735
FFM_T3 (kg)	27.38	8.93	30.27	9.53	29.86	9.49	0.023	29.5	9.72	30.27	9.37	29.89	9.54	0.398
FM_T3 (kg)	27.2	10	27.83	10.12	27.74	10.1	0.645	26.11	9.58	29.14	10.48	27.64	10.15	0.002
BF_T3 (%)	49.55	15.2	47.69	15.14	47.95	15.15	0.362	46.92	15.84	48.72	14.62	47.83	15.24	0.212
FFM_T1-T3_week_gain (g)	−0.52	0.42	−0.46	0.46	−0.47	0.45	0.372	−0.46	0.46	−0.47	0.44	−0.47	0.45	0.648
FM_T1_T3_week_gain (g)	0.85	0.46	0.82	0.47	0.83	0.46	0.607	0.79	0.48	0.85	0.45	0.82	0.46	0.243
%BF_T1-T3_week_gain (g)	1.47	0.87	1.32	0.85	1.34	0.86	0.199	1.35	0.9	1.32	0.82	1.33	0.86	0.74

Means were compared using independent sample *t*-test. T1, T2 and T3 corresponds to Trimester 1, 2 and 3; % BF, body fat percentage; T1-T3_week_gain (g), average weekly gain in g between Trimester 1 and 2.

**Table 6:** Univariate and multivariate logistic regression analysis of predictors for low birth weight and small for gestational age.

Parameters	Low Birth Weight						Small for Gestational Age					
	COR	95% CI	*p*	AOR ^#^	95% CI	*p*	COR	95% CI	*p*	AOR ^$^	95% CI	*p*
Neonatal sex												
Male	1			1			1			1		
Female	1.57	0.999–2.46	0.051	1.75	1.06–2.87	0.027	0.84	0.61–1.16	0.3	0.85	0.61–1.18	0.327
Gestational age												
Term	1			1								
Preterm	29.06	8.09–104.38	<0.000	28.66	7.63–107.72	0.00						
Mother’s age												
>30	1			1			1			1		
20–29	0.29	0.11–0.75	0.011	0.40	0.11–1.44	0.16	1.29	0.51–3.27	0.58	1.43	0.53–3.87	0.48
<20	0.43	0.15–1.28	0.132	0.42	0.099–1.78	0.24	1.33	0.48–3.71	0.57	1.48	0.49–4.44	0.48
Mother’s BMI at T1												
Normal	1			1			1			1		
CED	1.78	1.012–3.12	0.045	1.68	0.91–3.11	0.09	1.3	0.86–1.99	0.20	1.3	0.88–2.08	0.15
Overweight	1.4	0.80–2.57	0.226	1.36	0.717–2.57	0.36	0.98	0.64–1.49	0.93	1.04	0.67–1.59	0.86
Mother’s education												
>Higher	1			1			1			1		
secondary	1.8	1.07–3.03	0.026	1.4	0.78–2.56	0.25	1.25	0.87–1.80	0.23	1.17	0.79–1.73	0.41

COR, crude odds ratio; AOR, adjusted odds ratio; CI, confidence interval; CED, chronic energy deficient; BMI, body mass index; T1, Trimester 1. ^#^, models adjusted for neonatal sex, gestational age, maternal age, maternal BMI at Trimester 1, maternal education and family income; ^$^, model as done for LBW, except gestational age was not adjusted again for SGA.

## Data Availability

Not applicable.

## Supplementary Materials

The following supporting information can be downloaded at: https://www.mdpi.com/article/10.3390/children9101460/s1, Table S1: Median Intake of Food Groups by Pregnant Women and Its Implications on Birth Weight and Small for Gestational Age; Table S2: Birth weight and small for gestational age based on maternal macro and micronutrient intakes; Table S3: Birth weight and Small for Gestational age of Children based on Intake of Food group tertiles.
