# Supplementary material for: Maternal Nutrition, Body Composition and Gestational Weight Gain on Low Birth Weight and Small for Gestational Age—A Cohort Study in an Indian Urban Slum
Source: Children (Basel). 2022 Sep 23;9(10):1460. doi: 10.3390/children9101460 (PMC9600910; doi:10.3390/children9101460)
Supplement: Supplementary file 1 [file children-09-01460-s001.zip › children-1881235-supplementary.pdf]

Supplementary _files

Supple Table-S1: Median Intake of Food Groups by Pregnant Women and Its Implications on Birth Weight and Small for Gestational Age

Food groups	RDA 2020	Birth Weight				*p*	Weight for gestational age				*p*
		LBW		NBW			SGA		AGA		
		Median	IQR	Median	IQR		Median	IQR	Median	IQR	
Cereals and Millets (g)	325	361.77	234.11	376.47	230.24	0.508	376.89	230.26	368.85	227.09	0.519
Pulses and Legumes (g)	90	24.29	66.08	23.48	64.19	0.503	22.4	63.22	23.19	63.3	0.807
Meat, Poultry, Fish and Sea foods (g)	50	0.24	44.05	26.03	50	0.252	11.2	50	26.03	51.74	0.164
Fats and Edible Oils (g)	25	20.73	20.32	21.36	19.73	0.84	21.35	19.7	21.57	19.37	0.609
Milk and Milk Products (g)	400	224.7	193.03	231.48	255.78	0.47	217.6	238.36	238.7	254.12	0.458
Nuts and Oil seeds (g)	40	2.78	16.62	2.95	16.62	0.914	2.7	14.91	3.3	20.84	0.411
Green leafy and other Vegetables (g)	300	49.18	113.22	31.21	81.94	0.094	36.85	85.79	30.39	85.66	0.145
Roots and tubers (g)	100	33.37	44.01	30.06	49.84	0.739	31.89	45.01	28.7	51.62	0.473
Fruits (g)	150	75.32	104.67	76.76	117.4	0.544	75.63	112	74.81	124.9	0.521

RDA. Recommended Dietary Allowance; LBW, low birth weight; NBW, normal birth weight; SGA, small for gestational age; AGA, appropriate for gestational age. Groups were compared using Mann-Whitney U-test. Birth weight <2.5 kg=LBW. Infant birth weight <10 percentiles for the gestational age and gender=SGA

Supple Table S2: Birth weight and small for gestational age based on maternal macro and micronutrient intakes

Macro and Micro Nutrients	EAR 2020	Birth Weight				*p* value	Weight for Gestational Age				*p* value
		LBW		Normal			AGA		SGA		
		Median	IQR	Median	IQR		Median	IQR	Median	IQR	
Energy (Kcal)	2480	2310.9	929.87	2418.5	1216.8	0.282	2369.24	1204.47	2403.48	1123.39	0.858
Protein (g)	54	62.32	33.46	64.37	37.81	0.396	64.05	36.82	64.65	36.34	0.937
Total Fat (g)	30	51.6	33.46	56.39	41.05	0.621	57.63	44.30	52.33	33.60	0.371
Thiamine (mg)	1.6	1.04	.66	1.06	.65	0.470	1.04	.64	1.07	.65	0.662
Riboflavin (mg)	2.3	0.89	.46	0.89	.49	0.523	0.89	.46	0.89	.50	0.982
Niacin (mg)	14	9.98	6.87	10.76	6.52	0.405	10.2	6.37	10.8	6.38	0.663
Dietary folate (µg)	480	217.5	168.88	191.38	139.00	0.552	190.96	145.82	197.56	138.73	0.856
Vitamin C (mg)	65	45.54	49.22	45.99	44.86	0.807	45.99	46.46	44.8	43.94	0.382
Vitamin A (µg)	406	195.96	247.72	202.39	217.84	0.959	202.11	222.88	201.56	215.23	0.378
Iron (mg)	21	10.32	6.41	9.91	6.72	0.513	9.72	6.81	10.27	6.55	0.599
Zinc (mg)	12	8.02	4.53	8.05	4.96	0.600	7.64	5.07	8.15	4.33	0.58
Calcium (mg)	800	543.54	385.09	494.07	360.15	0.544	511.05	353.79	495.08	363.28	0.77
Groups were compared using Mann-Whitney U-test											

Supple Table S3: Birth weight and Small for Gestational age of Children based on Intake of Food group tertiles

		Birth Weight							Weight for gestational age						
		LBW		NBW		Total			AGA		SGA		Total		
		n	%	n	%	n	%	*p* value	n	%	n	%	n	%	*p* value
Cereals and Millets	1st Tertile(<307)	27	34.6	135	32.1	162	32.5	0.831	83	34.0	77	31.7	160	32.9	0.88
	2nd Tertile(307-455)	27	34.6	145	34.5	172	34.5		82	33.6	87	35.8	169	34.7	
	3rd Tertile(≥456)	24	30.8	140	33.3	164	32.9		79	32.4	79	32.5	158	32.4	
Pulses and Legumes	1st Tertile(<5)	22	28.2	139	33.1	161	32.3	0.852	79	32.4	80	32.9	159	32.6	0.662
	2nd Tertile(5-49)	29	37.2	139	33.1	168	33.7		86	35.2	80	32.9	166	34.1	
	3rd Tertile(≥50)	27	34.6	142	33.8	169	33.9		79	32.4	83	34.2	162	33.3	
Fats and Edible Oils	1st Tertile(<16)	26	33.3	138	32.9	164	32.9	0.671	76	31.1	84	34.6	160	32.9	0.538
	2nd Tertile(16-27)	30	38.5	139	33.1	169	33.9		83	34.0	82	33.7	165	33.9	
	3rd Tertile(≥28)	22	28.2	143	34.0	165	33.1		85	34.8	77	31.7	162	33.3	
Milk and Milk Products	1st Tertile(<144)	26	33.3	134	31.9	160	32.1	0.945	80	32.8	79	32.5	159	32.6	0.553
	2nd Tertile(144-292)	30	38.5	142	33.8	172	34.5		82	33.6	85	35.0	167	34.3	
	3rd Tertile(≥293)	22	28.2	144	34.3	166	33.3		82	33.6	79	32.5	161	33.1	
Nuts and Oil seeds	1st Tertile(<1)	34	43.6	155	36.9	189	38.0	0.79	89	36.5	95	39.1	184	37.8	0.291
	2nd Tertile(1-7)	16	20.5	121	28.8	137	27.5		69	28.3	63	25.9	132	27.1	
	3rd Tertile(≥8)	28	35.9	144	34.3	172	34.5		86	35.2	85	35.0	171	35.1	
Vegetables	1st Tertile(<12)	23	29.5	138	32.9	161	32.3	0.257	87	35.7	70	28.8	157	32.2	0.147
	2nd Tertile(12-68)	22	28.2	151	36.0	173	34.7		78	32.0	89	36.6	167	34.3	
	3rd Tertile(≥69)	33	42.3	131	31.2	164	32.9		79	32.4	84	34.6	163	33.5	
Roots and tubers	1st Tertile(<18)	25	32.1	138	32.9	163	32.7	0.157	89	36.5	70	28.8	159	32.6	0.65
	2nd Tertile(18-45)	29	37.2	135	32.1	164	32.9		74	30.3	89	36.6	163	33.5	
	3rd Tertile(≥46)	24	30.8	147	35.0	171	34.3		81	33.2	84	34.6	165	33.9	
Fruits	1st Tertile(<42)	28	35.9	137	32.6	165	33.1	0.549	84	34.4	79	32.5	163	33.5	0.809
	2nd Tertile(42-119)	26	33.3	140	33.3	166	33.3		76	31.1	87	35.8	163	33.5	
	3rd Tertile(≥120)	24	30.8	143	34.0	167	33.5		84	34.4	77	31.7	161	33.1	
Groups were compared based on categories using Chi-square test															
